# Time-course changes in DNA damage of corneal epithelial cells in rabbits following ocular instillation with genotoxic compounds

**DOI:** 10.1186/s41021-022-00243-4

**Published:** 2022-05-09

**Authors:** Haruna Tahara, Yoshinori Yamagiwa, Yu Haranosono, Masaaki Kurata

**Affiliations:** Research & Development Division, Senju Pharmaceutical Co., Ltd., 6-4-3, Minatojima-Minamimachi, Chuo-Ku, Kobe, Hyogo 650-0047 Japan

**Keywords:** Comet assay, Rabbit, Corneal epithelial cells, Genotoxicity test, Ophthalmic drug, Sampling time

## Abstract

**Background:**

In eye-drop drug development, the additional genotoxicity tests in some cases might be necessary to assess genotoxicity in the ocular surface since the ocular surface is exposed directly to high drug concentrations. Recently, an in vivo comet assay using corneal epithelial cells in rabbits following single ocular instillation was developed as an assay to evaluate genotoxicity in ocular tissues. In this study, we investigated the time-course changes in DNA damage after ocular instillation of genotoxic compounds to evaluate the optimal sampling timing for in vivo comet assay of the ocular surface tissue. Ethidium bromide (EtBr), methyl methanesulfonate (MMS), and 4-nitroquinoline 1-oxide (4-NQO) were administered to the eyes of the rabbits. Corneas were collected at 0.5, 2, 4, 6, and 24 h after administration, and the comet assay was performed. In addition, the in vitro comet assay was performed to assess the time-course changes in DNA damage induced by short-time exposure to the genotoxic compounds.

**Results:**

The mean % tail DNA, which is an indicator for DNA damage, in the corneal epithelial cells treated with all compounds exhibited statistically significant increases compared with those in the negative controls of saline at 0.5, 2, 4, and 6 h. There was a difference in the DNA damage response between EtBr and the other two compounds. In the 3% MMS- and 1% 4-NQO-treated eyes, the values of the % tail DNA were the highest at 0.5 h and then decreased gradually. In contrast, in the 1% EtBr-treated eyes, the highest value was noted at 4 h. The results of the in vitro comet assay showed that the % tail DNA increased in all groups. A further increase in the % tail DNA occurred in the EtBr-treated cells even after removing the compound but not in the MMS- and 4-NQO-treated cells.

**Conclusion:**

Relatively high values of the % tail DNA were maintained from 0.5 to 6 h after administration, suggesting that the optimal sampling time is any one point from 0.5 to 6 h in the comet assay of the corneal surface.

## Introduction

The ophthalmic solution is applied directly to the surface of the eyes with a high active ingredient concentration [1]. Therefore, when developing ophthalmic drugs, it is important to evaluate the toxic effects on the administration site, the eyes, together with the systemic side effects. The genotoxicity test is one of the key non-clinical toxicity tests, as genotoxicity can result in carcinogenesis. However, to date, there have been few reports on genotoxicity tests on the ocular surface after ocular instillation. In our previous study of the in vivo comet assay using rabbit corneas, DNA damage was detected in some genotoxic compounds after 2 h of single ocular instillation [2]; however, it remains unclear whether the corneal sampling time of 2 h is optimal.

The comet assay for systemic drugs guideline (OECD TG 489) describes that the sampling time in the systemic tissues should be determined based on pharmacokinetic data (e.g., time to reach maximum plasma or tissue concentration [T_max_]) [3]. In addition, the guideline mentions that, in the absence of pharmacokinetic data, the sampling timing should be 2–6 h after the last administration of repeated administration, or both 2–6 h and 16–26 h after a single administration. In in vivo comet assays using the liver and stomach, the tissue sampling timing is often 3 h (and 24 h) post-administration [4]. In ocular instillation, T_max_ in the ocular tissues may be faster than in systemic tissues such as the liver because the ophthalmic solution is exposed directly to the ocular surface. Therefore, it is necessary to set the optimal sampling time for evaluating genotoxicity on the ocular surface.

In this study, we investigated the time-course change of DNA damage in corneal epithelial cells of rabbits after single ocular instillation to obtain optimal tissue sampling timing. The comet assay was performed at 0.5, 2, 4, 6, and 24 h after administration of the genotoxic compounds. We selected rabbits for this study because they are commonly used in ocular toxicity studies in drug developments [1, 5] and have been employed for the in vivo corneal comet assay [2]. In addition, we performed the in vitro comet assay using human corneal epithelial-transformed (HCE-T) cells that had been exposed to the genotoxic compounds for 1 min, and further cultured for 2, 4, 6, or 24 h after removing the compounds in order to compare with the results of the in vivo study. We used well-known genotoxic compounds: ethidium bromide (EtBr) for DNA intercalation [6], methyl methanesulfonate (MMS) for alkylation [7], and 4-nitroquinoline 1-oxide (4-NQO) for bulky DNA adduct formation [8].

## Materials and methods

### Chemicals

EtBr (CAS No. 1239-45-8), MMS (CAS No. 66–27-3), and 4-NQO (CAS No. 56–57-5) were used as test compounds. EtBr was purchased from Nacalai Tesque, Inc. (Kyoto, Japan), and MMS and 4-NQO were purchased from FUJIFILM Wako Pure Chemical Corporation (Osaka, Japan). Saline and dimethyl sulfoxide (DMSO) were obtained from Otsuka Pharmaceutical Factory, Inc. (Tokushima, Japan) and Nacalai Tesque, Inc., respectively.

### Animals and husbandry

Male Japanese white rabbits (Kbs:JW) of 9–12 weeks of age were purchased from Kitayama Labes, Co., Ltd. (Nagano, Japan), and the test compounds were administered at 11–15 weeks of age (bodyweight 2.0–2.7 kg). The rabbits were individually housed in air-conditioned rooms with 19–25 °C temperature, 40–70% relative humidity, and a 12-h light/dark cycle. Each rabbit was provided with commercial pellet feed (LRC4; Oriental Yeast Co., Ltd., Tokyo, Japan) and tap water ad libitum. The dumbbell made from polypropylene and wood gnawing block (Bio-Serv, Flemington, NJ) were placed in each cage as the environmental enrichment devices. The animals acclimated for at least 6 days before the experiments. Animal care and treatment were provided in accordance with the standard procedures of the facility, which are fully accredited by the Association for Assessment and Accreditation of Laboratory Animal Care International. All experimental procedures were in accordance with the guidelines for animal experimentation at Senju Pharmaceutical Co., Ltd., and the protocol was reviewed by the Institutional Animal Care and Use Committee of Senju Pharmaceutical Co., Ltd.

### Procedures for animal treatments

**Fig. 1:**
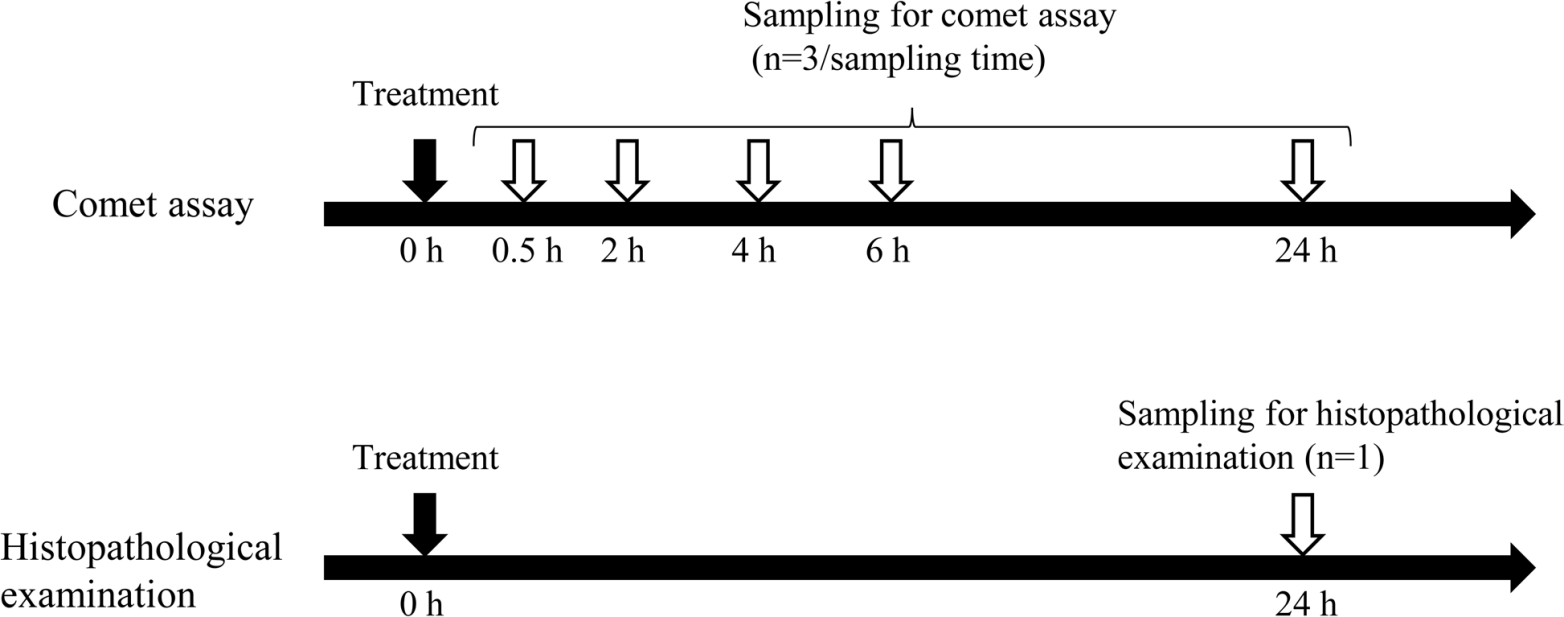
Treatment schedule. Comet assay of corneal epithelial cells in rabbit was performed 0.5, 2, 4, 6, and 24 h after ocular instillation of the test compounds. Histopathological examination of the rabbit eyes was performed 24 h after administration. The experiments were performed per sampling time

The study schedule is shown in Fig. 1. Experiments were performed per sampling time, and 32 clinically normal rabbits were randomly assigned to each treatment group. To prepare the dosing solutions, EtBr and MMS were dissolved in saline, and 4-NQO was suspended in 5% DMSO in saline. Based on the in vivo corneal comet assay results [2], the concentrations that do not cause irritative or histopathological changes in the eye were set. Saline was used as a negative control in this study because the value of the % tail DNA in the 5% DMSO-treated cells (7.9%) was within the range of that in the saline-treated cells (7.3–8.8%) in the previously performed corneal comet assay in rabbits [2].

For comet assay, 50 μL of saline or 3% MMS (1.5 mg/eye) was instilled once per right eye of 3 rabbits per group, and 50 μL of 1% EtBr (0.5 mg/eye) or 1% 4-NQO (0.5 mg/eye) was administered to the left eyes in the same manner (three eyes per group). After ocular instillation, the eyelids were artificially blinked several times. The rabbits were euthanized after 0.5, 2, 4, 6, and 24 h by intravenously administering an overdose (approximately 90 mg/kg) of thiopental solution (Ravonal; Nipro ES Pharma Co. Ltd., Osaka, Japan). The eyeballs of the rabbits were collected after euthanasia and subjected to the comet assay.

For histopathological examination, 50 μL of saline or 3% MMS was instilled once onto the right eyes of one rabbit per group, and 50 μL of 1% EtBr or 1% 4-NQO was administered to the left eye in the same manner as the comet assay (one eye per group). Twenty-four hours after administration, the anterior segment of the eye was observed using a slit-lamp biomicroscope SL-130 (Carl Zeiss Meditec AG, Jena, Germany). The corneal epithelial damage was evaluated by staining with fluorescein (AYUMI Pharmaceutical Corporation, Tokyo, Japan). After anterior ocular observation, the rabbits were euthanized in the same manner as the comet assay, and the eyeballs of the rabbits were collected.

### Isolation of corneal epithelial cells for the comet assay

The collected eyes were washed with Ca^2+^- and Mg^2+^-free phosphate-buffered saline (PBS). After washing, the corneas were removed from the eyes using scissors. The removed corneas were treated with 1.2 unit/mL Dispase II (Roche Diagnostics K.K., Tokyo, Japan) in minimum essential media (MEM; Thermo Fisher Scientific K. K., Tokyo, Japan) supplemented with 10% bovine serum (10% BS/MEM) overnight at 4 °C. Corneal epithelial cells were isolated using a spatula and placed into fresh 10% BS/MEM. The cells were centrifuged at 200×*g* for 5 min, and the supernatants were discarded. The cells were resuspended in 1 mL of 0.25% trypsin (Thermo Fisher Scientific K. K.) and incubated for 10 min at 37 °C. Furthermore, 8 mL of 10% BS/MEM was added to the cells, and the cell suspensions were passed through a 70-μm cell strainer. The cells were centrifuged at 200×*g* for 5 min, and the supernatants were discarded. The cells were then resuspended with PBS to a density of approximately 2 × 10^5^ cells/mL.

### Alkaline comet assay

The alkaline comet assay was conducted according to previously published method [2]. A 30 μL of the prepared cell suspension was mixed with 270 μL of melted agarose solution (CometAssay LMAgarose; Trevigen, Inc., Gaithersburg, MD). Then, 30 μL of this mixture was placed in each well of a 20-well slide (CometSlide HT, Trevigen, Inc.), and the slide was left for approximately 10 min at 4 °C to solidify the agarose. The slide was immersed in lysis solution (pH 10) containing 2.5 M sodium chloride, 100 mM di-sodium dihydrogen ethylenediaminetetraacetate dihydrate (EDTA∙2Na), 10 mM tris(hydroxymethyl)aminomethane (Tris), and 1% (v/v) polyethylene glycol mono-*p*-isooctylphenyl ether for 1 h at 4 °C. Subsequently, the slide was immersed in an alkaline unwinding solution (200 mM sodium hydroxide and 1 mM EDTA, pH > 13) for 20 min at room temperature. Electrophoresis was performed with the same solution at 1 V/cm for 30 min under refrigeration. After electrophoresis, the slide was washed twice with ultrapure water and dehydrated by immersion in ethanol for 10 min. The slide was stained with SYBR Green I Nucleic Acid Gel Stain (excitation maxima at 497 nm, emission maxima at 520 nm; Thermo Fisher Scientific K. K.) diluted 1:1000 with Tris-EDTA buffer (pH 7.5) and mounted using ProLong Gold (Thermo Fisher Scientific K. K.). The slide was observed using a BX51 fluorescence microscope (Olympus Corporation, Tokyo, Japan) with a NIBA filter (excitation at 460–495 nm and emission at 510–550 nm) equipped with a CCD camera (scA1300-32 fm; Basler AG, Ahrensburg, Germany).

First, the number of “hedgehogs” was counted among 150 comets per eye (450 comets per group). According to the Atlas of Comet Assay Images, hedgehogs are highly fragmented cells presenting as a small or non-existent comet head and large diffuse comet tail [9]. Second, 150 scorable comets (i.e., with a clearly defined head and tail with no interference from neighboring cells) without hedgehogs were measured per eye (450 comets per group). The % tail DNA (DNA fluorescence intensity in the tail/total DNA fluorescence intensity × 100) was measured as a DNA damage indicator using the Comet Assay IV software, version 4.3.2 (Perceptive Instruments, Haverhill, UK).

### Microscopic examination of the corneal tissue

The collected eyes (one eye per group) were fixed with 1% formaldehyde/2.5% glutaraldehyde in 0.1 M phosphate buffer fixative overnight at 4 °C and post-fixed with 10% neutral-buffered formalin solution. The tissues were dehydrated using a graded alcohol series and embedded in paraffin. Approximately 3-μm-thick corneal tissue sections were prepared and stained with hematoxylin and eosin. All microscopic images were obtained with a BX53 microscope fitted with a DP74 digital camera (Olympus Corporation) and analyzed using the cellSens Standard imaging software, version 2.3 (Olympus Corporation).

### Statistical analysis

The mean and standard deviation (SD) of the % tail DNA and the number of hedgehogs were calculated for all experimental groups. The median % tail DNA was also calculated according to the OECD TG 489 [3]. The data were assumed to have a normal distribution and homogeneous variance. For each corneal sampling time, Student’s *t*-test (one-tailed) was used to compare the mean of each test compound group with that of the negative control group for % tail DNA and hedgehog. JMP version 15.1.0 (SAS Institute Japan, Ltd., Tokyo, Japan) was used for all statistical analyses. Probability (*p*) values of < 0.05 were considered statistically significant.

### Measurement of DNA damage and cell viability in vitro

To assess time-course changes in DNA damage after short-time exposure to the genotoxic compounds, the in vitro comet assay was performed using HCE-T cells with reference to our previous study [10]. Briefly, the HCE-T cells were seeded in a 12-well culture plate at a density of 5 × 10^4^ cells/well (1 mL/well) and incubated for approximately 24 h. After a 24-h incubation, the cells were washed with PBS. The cells were then exposed to a mixture of 500 μL of serum-free medium and an equal volume of the test compounds which were dissolved in distilled water (Otsuka Pharmaceutical Factory, Inc.) at twice concentration of final concentration (final concentration: 0.5% EtBr, 0.5% MMS, and 0.001% 4-NQO) for 1 min at room temperature. After the treatment, the cells were washed with PBS, and 1 mL of fresh culture medium was added. The cells were further incubated for 2, 4, 6, and 24 h at 37 °C. After incubation, the cells were trypsinized (TrypLE™ Express, Thermo Fisher Scientific K.K.). In the same manner, the cells were exposed to the test compounds or distilled water as a negative control for 1 min to evaluate DNA damage immediately after exposure. Immediately after the treatment, the cells were washed and trypsinized. The collected cells were centrifuged and re-suspended in PBS at a density of 1–2 × 10^5^ cells/mL. Comet assay was performed as described previously herein (*n* = 3/group).

For cytotoxicity assay, the HCE-T cells were seeded in a 96-well culture plate at a density of 5 × 10^3^ cells/well (100 μL/well) and incubated for approximately 24 h. After the 24-h incubation, the cells were washed with PBS. The cells were then exposed to a mixture of 50 μL of serum-free medium and an equal volume of test compounds at twice concentration of final concentration for 1 min at room temperature. After the treatment, the cells were washed with PBS, and 100 μL of fresh culture medium was added. The cells were further incubated for 2, 4, 6, and 24 h at 37 °C. After that, the cells were incubated with 100 μL of Cell Counting Kit-8 (Dojindo Laboratories Co., Ltd., Kumamoto, Japan) diluted to one-tenth with a serum-free medium for 2 h at 37 °C. Absorbance was measured at 450 nm using a microplate reader (Synergy HTX, BioTek Instruments Inc., Winooski, VT). Cell viability was calculated as follows: the absorbance of compound-treated cells/the absorbance of non-treated cells, expressed as a percentage (*n* = 3/group).

The mean and SD of the % tail DNA and cell viability (%) were calculated for all experimental groups. For % tail DNA, Dunnett’s multiple comparison test (one-tailed) was used to compare the mean of each test compound group with that of the negative control group. For cell viability (%), Dunnett’s multiple comparison test (one-tailed) was used to compare the mean of each test compound group with that of the non-treated group. JMP version 15.1.0 was used for all statistical analyses. Probability (*p*) values of < 0.05 were considered statistically significant.

## Results

### Comet assay of corneal epithelial cells

Table 1 shows the time-course changes in the % tail DNA for the three genotoxic compounds. In the saline-treated group, the mean % tail DNA was approximately 10% throughout sampling times of 0.5, 2, 4, 6, and 24 h. In the 1% EtBr-treated group, increases in the % tail DNA values at 0.5, 2, 4, and 6 h were statistically significant compared with those in the saline-treated group. The values were approximately 30% at 0.5 and 2 h and reached the highest value at 4 h, which slightly decreased at 6 h, resulting in a similar value as that of the saline-treated group at 24 h. In the 3% MMS- and 1% 4-NQO-treated groups, statistically significant increases in the % tail DNA values were observed at all sampling times compared with those in the saline-treated group. The values were the highest at 0.5 h and then gradually decreased with time; however, the values were not as low as that of the saline-treated group, even after 24 h. The increases in hedgehog were observed in all test compounds, along with increasing in the % tail DNA (Table 2). In the EtBr- and 4-NQO-treated groups, the hedgehog frequency significantly increased at 4 h, and both 0.5 h and 24 h, respectively, compared with those in the saline-treated group. In the MMS-treated group, statistically significant increases were observed at all sampling times compared with that of the saline-treated group.

**Table 1:** Time-course changes of the % tail DNA in the corneal epithelial cells of rabbit

Compounds	% tail DNA (%)				
	0.5 h	2 h	4 h	6 h	24 h
Saline	9.6 ± 4.8 (5.0)	13.4 ± 1.0 (8.3)	10.2 ± 1.3 (5.7)	9.8 ± 3.1 (3.2)	8.3 ± 1.7 (2.6)
1% EtBr	31.0 ± 3.0^a^ (25.8)	28.1 ± 1.6^a^ (22.6)	55.3 ± 1.4^a^ (61.5)	41.0 ± 6.5^a^ (40.4)	10.0 ± 4.0 (5.4)
3% MMS	66.2 ± 1.8^a^ (70.8)	63.6 ± 4.4^a^ (69.4)	47.1 ± 7.2^a^ (48.9)	50.1 ± 2.8^a^ (56.0)	40.1 ± 2.4^a^ (38.1)
1% 4-NQO	55.2 ± 11.2^a^ (58.3)	38.9 ± 7.8^a^ (39.4)	35.9 ± 2.6^a^ (36.0)	26.1 ± 2.6^a^ (22.0)	18.7 ± 5.0^a^ (14.5)

Data are presented as the mean ± SD (*n* = 3). Parentheses indicate the median

*EtBr* Ethidium bromide, *MMS* Methyl methanesulfonate, *4-NQO* 4-nitroquinoline 1-oxide

^a^Significantly higher than the negative control group at 5% probability level (Student’s *t*-test, one-tailed)

**Table 2:** Time-course changes in the hedgehog frequency in the corneal epithelial cells of rabbit

Compounds	Frequency of hedgehog (%)				
	0.5 h	2 h	4 h	6 h	24 h
Saline	0.2 ± 0.6	1.1 ± 1.2	0.7 ± 1.0	0.7 ± 0.0	0.4 ± 0.6
1% EtBr	3.6 ± 2.5	3.3 ± 1.7	8.9 ± 3.5^a^	1.6 ± 2.3	0.4 ± 0.6
3% MMS	12.9 ± 7.8^a^	13.6 ± 8.3^a^	10.7 ± 1.0^a^	8.0 ± 2.0^a^	2.0 ± 1.0^a^
1% 4-NQO	9.8 ± 8.5^a^	3.8 ± 2.9	1.3 ± 1.0	1.1 ± 0.6	2.0 ± 2.0^a^

Data are presented as the mean ± SD (*n* = 3)

*EtBr* Ethidium bromide, *MMS* Methyl methanesulfonate, *4-NQO* 4-nitroquinoline 1-oxide

^a^Significantly higher than the negative control group at 5% probability level (Student’s *t*-test, one-tailed)

**Fig. 2:**
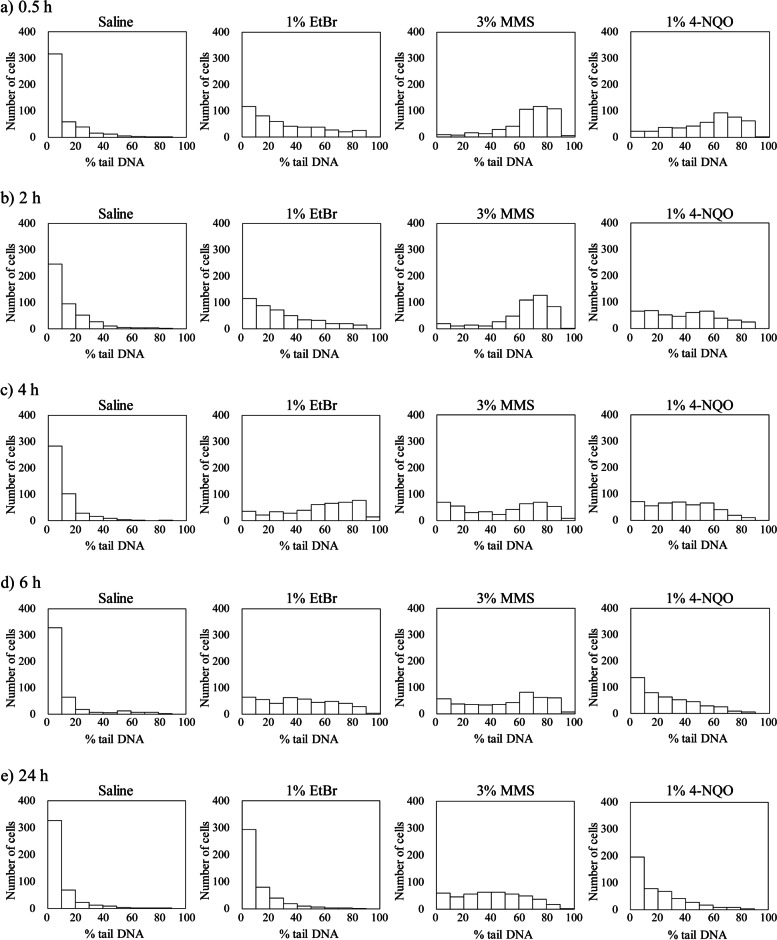
Distribution of the % tail DNA in the corneal epithelial cells. Saline as the negative control, 1% ethidium bromide (EtBr), 3% methyl methanesulfonate (MMS), or 1% 4-nitroquinoline 1-oxide (4-NQO) was administered once to the eyes of the rabbits. The corneas were collected 0.5 (**a**), 2 (**b**), 4 (**c**), 6 (**d**), and 24 h (**e**) after administration, and the comet assay was performed using the corneal epithelial cells. Data represents the results for 450 cells per group

Figure 2 shows the distribution of the % tail DNA values in corneal epithelial cells in pooled data from three eyes. In the saline-treated group, most cells showed values within the range of 0 and 20% at all sampling times. In the test compound-treated groups, the distributions were shifted to the right (i.e.*,* high value) at all sampling times, except the EtBr-treated group at 24 h. In the EtBr-treated group, the distribution was relatively similar to that of saline-treated group at 24 h.

### Ophthalmological examination of anterior ocular segment

Twenty-four hours after administration, minimal swelling in the conjunctiva, slight hyperemia of the iris, and slight fluorescein staining in the corneal epithelium were observed in the 1% 4-NQO-treated eye. No abnormal ophthalmological changes were observed in the 1% EtBr-, 3% MMS-, and saline-treated eyes.

### Histopathological examination of cornea

**Fig. 3:**
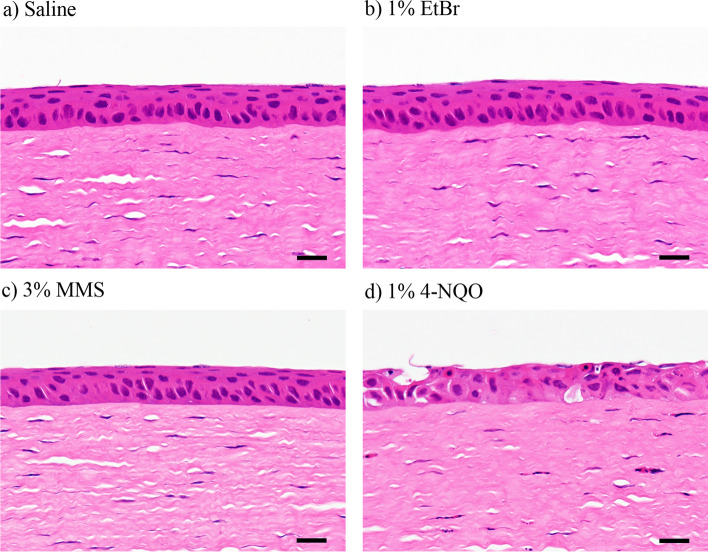
Representative photomicrographs of the corneal epitheliums in the rabbits. Saline as the negative control, 1% ethidium bromide (EtBr), 3% methyl methanesulfonate (MMS), or 1% 4-nitroquinoline 1-oxide (4-NQO) was administered once to the eyes of rabbits. The corneas were collected at 24 h, and corneal sections were stained with hematoxylin and eosin. No histopathological changes in the corneal epithelium were observed in the saline (**a**), 1% EtBr (**b**), and 3% MMS-treated eyes (**c**). Moderate degeneration/necrosis was noted in the corneal epithelium in the 1% 4-NQO-treated eyes (**d**). Scale bars: 20 μm

Table 3 summarizes the histopathological findings in the corneal epithelium at 24 h. No histopathological changes in the corneal epithelium were observed in the saline- and 1% EtBr-treated eyes (Fig. 3a and b). In the 3% MMS-treated eyes, no histopathological changes in the corneal epithelium were observed (Fig. 3c), but minimal infiltration of inflammatory cells was found in the corneal limbus. In the 1% 4-NQO-treated eyes, moderate degeneration/necrosis was observed in the corneal epithelium (Fig. 3d). In addition, minimal infiltration of inflammatory cells was noted in the corneal limbus and stroma.

**Table 3:** Histopathological findings in the rabbit cornea at 24 h after administration

Findings	Compounds			
	Saline	1% EtBr	3% MMS	1% 4-NQO
No. of corneas examined	1	1	1	1
Degeneration/necrosis, corneal epithelium	–	–	–	++
Infiltrate, inflammatory cell, corneal limbus	–	–	±	±
Infiltrate, inflammatory cell, corneal stroma	–	–	–	±

−: No abnormal findings, ±: minimal, ++: moderate

*EtBr* ethidium bromide, *MMS* methyl methanesulfonate, *4-NQO* 4-nitroquinoline 1-oxide

### In vitro comet assay and cytotoxicity assay

**Fig. 4:**
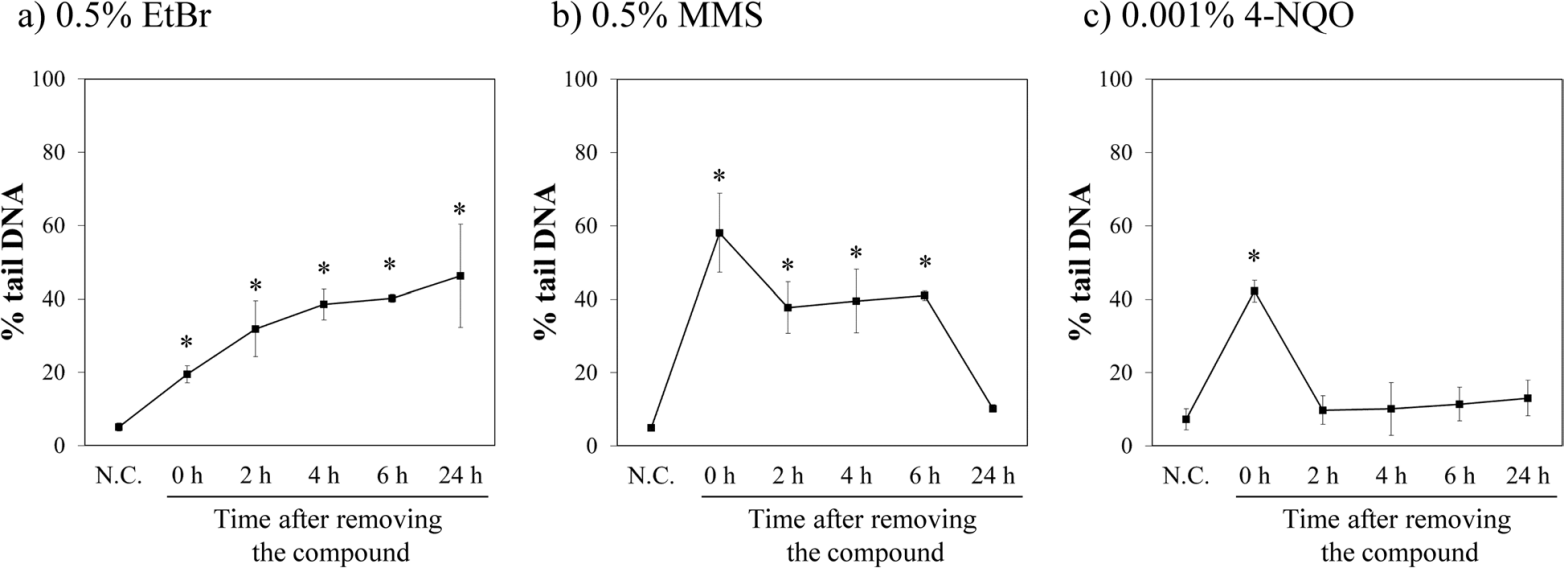
Change in % tail DNA in human corneal epithelial cells treated with genotoxic compounds. Human corneal epithelial-transformed (HCE-T) cells were exposed to three compounds (0.5% ethidium bromide, EtBr; 0.5% methyl methanesulfonate, MMS; and 0.001% 4-nitroquinoline 1-oxide, 4-NQO) for 1 min. After treatment, the cells were washed. Fresh culture medium was added, and the cells were further incubated for 2, 4, 6, and 24 h. After incubation, the cells were collected. HCE-T cells were exposed to the test compounds or distilled water as a negative control for 1 min to evaluate DNA damage immediately after exposure (“0 h” or “N.C.” in the figures). Immediately after the treatment, the cells were collected. Comet assay was performed using the collected cell suspensions. Data show the mean ± SD (*n* = 3). *: Significantly higher than the negative control group at 5% probability level (Dunnett’s multiple comparison test, one-tailed)

The results of the comet assay using the HCE-T cells are shown in Fig. 4. The % tail DNA increased immediately after 1 min of treatment with all the test compounds. The % tail DNA gradually increased even after removing the compound in the EtBr-treated group (Fig. 4a). In the MMS- and 4-NQO-treated groups, the % tail DNA decreased after removing these compounds (Fig. 4b and c). Moderate cytotoxicity was observed at 24 h after removing the compound in the EtBr-treated group. At other times, slight cytotoxicity was noted. In the MMS- and 4-NQO-treated groups, no cytotoxicity was observed at any time (Table 4).

**Table 4:** Change in viability of human epithelial cells treated with genotoxic compounds

Incubation time after removing the compounds^a^	Cell viability (%)		
	0.5% EtBr	0.5% MMS	0.001% 4-NQO
Non-treated	100.0 ± 2.8	100.0 ± 2.6	100.0 ± 1.5
2 h	89.3 ± 6.1^b^	115.1 ± 5.1	139.2 ± 2.0
4 h	74.7 ± 8.0^b^	108.1 ± 0.3	114.2 ± 14.5
6 h	73.1 ± 4.6^b^	108.2 ± 4.9	122.7 ± 24.2
24 h	39.1 ± 1.3^b^	108.5 ± 11.3	118.3 ± 13.9

Data are presented as the mean ± SD (*n* = 3)

*EtBr* Ethidium bromide, *MMS* Methyl methanesulfonate, *4-NQO* 4-nitroquinoline 1-oxide

^a^Time of incubation with fresh culture medium after a 1 min exposure to each compound

^b^Significantly lower than the non-treated group at 5% probability level (Dunnett’s multiple comparison test, one-tailed)

## Discussion

In this study, we investigated the time-course changes in DNA damage on the ocular surfaces after instillation of known genotoxic compounds to obtain optimal tissue sampling time. In all test compound-treated groups, the statistically significant increases in the % tail DNA in the corneal epithelial cells were observed at 0.5–6 h compared with those of the negative controls. From the reason, the optimal corneal sampling time is suggested to be any one point from 0.5 to 6 h after instillation.

The peak value of the % tail DNA was observed at 0.5 h in the MMS- and 4-NQO-treated groups. The T_max_ in the cornea after single ocular instillation is generally approximately 0.25–0.5 h [11–13], and the peaks of DNA damage in two compounds were almost the same as the reported T_max_. MMS causes DNA methylation, which is repaired by base excision repair [14]. It is known that 4-NQO forms the DNA adduct, which is repaired by nucleotide excision repair [15]. Single strand breaks occur during those excision repair process [14]; therefore, the alkaline comet assay can detect single-strand breaks as an increase in DNA migration. DNA repair response is considered to initiate immediately after DNA methylation and DNA adduct formation induced by MMS and 4-NQO because DNA migration increased after 0.5 h. In the in vitro test, each peak of DNA damage was observed immediately after treatment with MMS or 4-NQO. The results were consistent with those of the in vivo study. From the viewpoint of peak of DNA damage and T_max_ in the cornea, appropriate timing for the tissue sampling is considered to be 0.5–6 h. In addition, the results of this study imply that it may be better to set earlier sampling time compared with the description in OECD TG 489 test guideline (i.e. 2–6 h of sampling time).

However, the DNA damage response of EtBr differed from the other two compounds; i.e. the peak of % tail DNA after administration of EtBr was later than that of the other two compounds, and was observed at 4 h. The peak of DNA damage was not always correlated with T_max_ in the cornea as described above. It is difficult to accurately measure the tissue concentration of EtBr, because EtBr has the property of enhancing fluorescence intensity when intercalating to DNA. Therefore, actual concentration of EtBr in the cornea after ocular instillation could not be determined in this study. EtBr intercalates between DNA base pairs and may cause nicks for DNA strands by inhibition of excision repair processes [16]. Moreover, intercalating agents reversibly bind to DNA, and inhibit DNA and RNA polymerase competition for DNA binding, thereby inhibiting DNA replication and translation [16]. A possible cause of delay of DNA damage was that intercalated EtBr molecules kept in the cells, and affect other DNA sites before repair. A similar tendency was observed in the in vitro study; DNA damage gradually increased with time after removing EtBr and was observed even at 24 h after removal. Although DNA migration could have increased due to cytotoxicity, especially at 24 h after removing EtBr, the DNA damage response induced by EtBr was different from that induced by the other two compounds. The results of this study suggest that genotoxic mode of action affect timing of DNA damage in several cases.

In the 1% 4-NQO-treated group in the in vivo study, the % tail DNA values decreased at 24 h compared to that at 0.5 h in parallel with decreasing hedgehog frequency, even though moderate degeneration/necrosis of the corneal epithelium was noted at 24 h. Histopathological changes such as cell infiltration, apoptotic, and necrotic changes are associated with increased DNA migration [3]. In some studies, no increased DNA migration was observed despite the necrosis or apoptosis presence in the target organ because heavily damaged cells may have been lost during sample processing or electrophoresis [17]. In this study, DNA damage caused by 4-NQO progressed DNA repair or cell death in which heavily damaged cells may have been lost in Dispase II and trypsin treatment processes. From the above, the present study again stressed the importance of performing a histopathological examination as well as comet assay. In addition, no histopathological findings were observed at 2 h in the previous study, although DNA damage was observed [2]. This finding recommends an appropriate tissue sampling timing of 0.5–6 h as the tissues can be collected before histopathological change.

The International Council for Harmonisation of Technical Requirements for Pharmaceuticals for Human Use (ICH) S2(R1) describes the standard battery of genotoxicity test [18]. Two in vitro tests (a bacterial mutation test and a mammalian cell genotoxicity test) and one in vivo test are required as Option 1. If an in vitro test using mammalian cells is positive, additional in vivo tests are recommended [18]. Our method can be applied as an additional in vivo test when the result of in vitro test using mammalian cells is positive in the development of ophthalmic drugs. In addition, from the viewpoint of 3Rs, investigating the integration of in vivo corneal comet assay with eye irritation test or repeated ocular instillation toxicity study (a general toxicity study) is valuable. Further studies are necessary when using other animal species such as rats, dogs, and monkeys because time-course responses of DNA damage are different across organs and animal species [19]. Furthermore, the time courses in DNA damage of the HCE-T cells in the in vitro comet assay were correlated well with that of the in vivo comet assay in this study. Therefore, in vitro comet assay may be a useful tool for predicting time-course change in DNA damage of in vivo comet assay.

## Conclusion

Our data showed the DNA damage level in time-course in the corneal epithelial cells of the rabbit following single ocular instillation of the three genotoxic compounds. Since statistically significant increases in the % tail DNA were observed at 0.5–6 h in all test compound-treated groups, the optimal corneal sampling time is suggested to be any one point from 0.5 to 6 h after instillation.

## Data Availability

All data generated or analyzed in this study are included in this published article. All materials used in this study are described in the article.

## Abbreviations

EtBr: Ethidium bromide
MMS: Methyl methanesulfonate
4-NQO: 4-Nitroquinoline 1-oxide
T_max_: Time to reach maximum plasma or tissue concentration
HCE-T: Human corneal epithelial-transformed
DMSO: Dimethyl sulfoxide
PBS: Ca^2+^- and Mg^2+^-free phosphate-buffered saline
MEM: Minimum essential media
BS: Bovine serum
EDTA∙2Na: di-Sodium dihydrogen ethylenediaminetetraacetate dehydrate
Tris: Tris(hydroxymethyl)aminomethane
SD: Standard deviation
ICH: International Council for Harmonisation of Technical Requirements for Pharmaceuticals for Human Use
